# Supplementary material for: bglG Regulates the Heterogeneity Driven by the Acid Tolerance Response in Lacticaseibacillus paracasei L9
Source: Foods. 2023 Oct 30;12(21):3971. doi: 10.3390/foods12213971 (PMC10650579; doi:10.3390/foods12213971)
Supplement: Supplementary file 1 [file foods-12-03971-s001.zip › foods-2629817-supplementary.pdf]

Supplementary Material

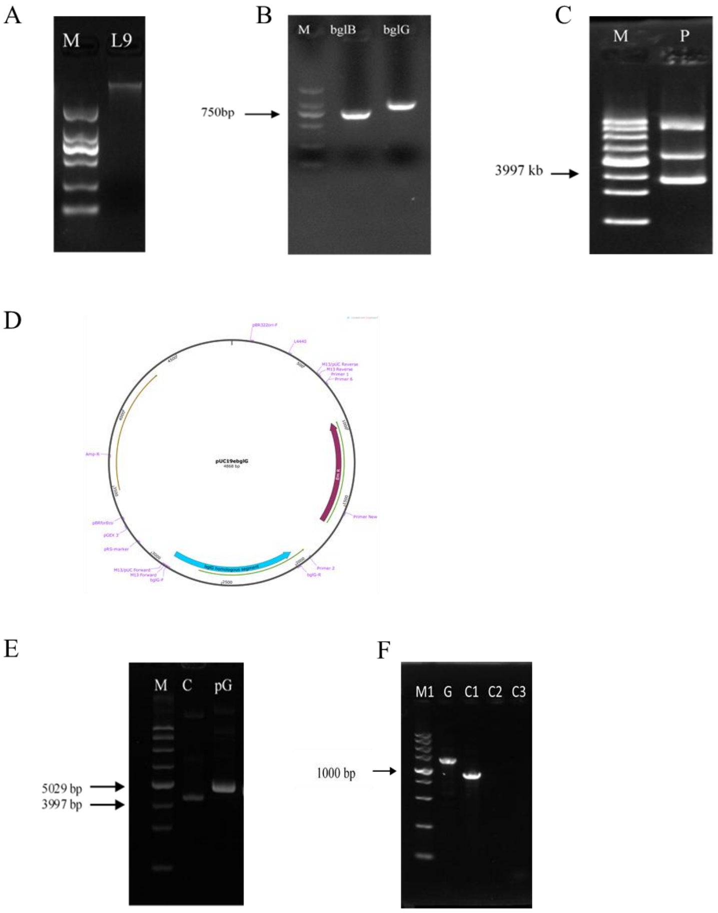

Figure S1. Insertional inactivation of *bglG*. (**A**) Agarose electrophoresis of the genomic DNA from *L. paracasei* L9. M: DNA marker D2000; L9: Genomic DNA from *L. paracasei* L9. (**B**) *bglG* homologous segment. M: DNA marker D2000; bglG: *bglG* homologous segment (predicted size 892 bp). (**C**) Plasmid pUC19e. M: Supercoiled DNA Ladder Marker; P: pUC19e. (**D-E**) Recombinant plasmids pUC19ebglG using T4DNA ligase to connect the homologous segment with the vector overnight. M: Supercoiled DNA Ladder Marker; C: pUC19e; pG: pUC19ebglG (predicted size 5000 bp); (**F**) Identification of the L9 insertion inactivated mutant. M1: DNA Marker DL5000; G: insert inactivated *bglG*- segment in L9*bglG*- (predicted size 1405 bp); C1: *bglG* homologous segment in L9*bglG*- (predicted size 892 bp); C2: blank control: C3: *bglG* homologous segment of wild-type L9.

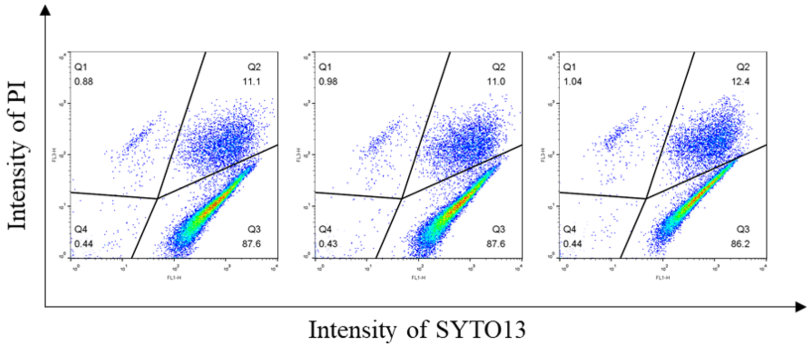

Subpopulation	Proportion（%）
FCM-V	87.1±0.7
FCM-I	11.5±0.6

Figure S2. Proportions of subpopulations induced by acid tolerance response of *L. paracasei* L9.

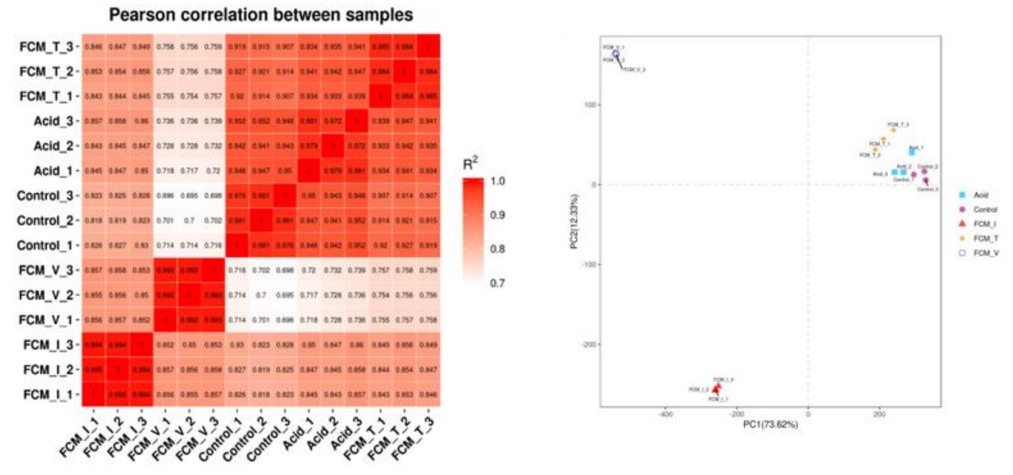

Figure S3. Pearson correlation and principal component analysis between samples of RNA-Sequencing.

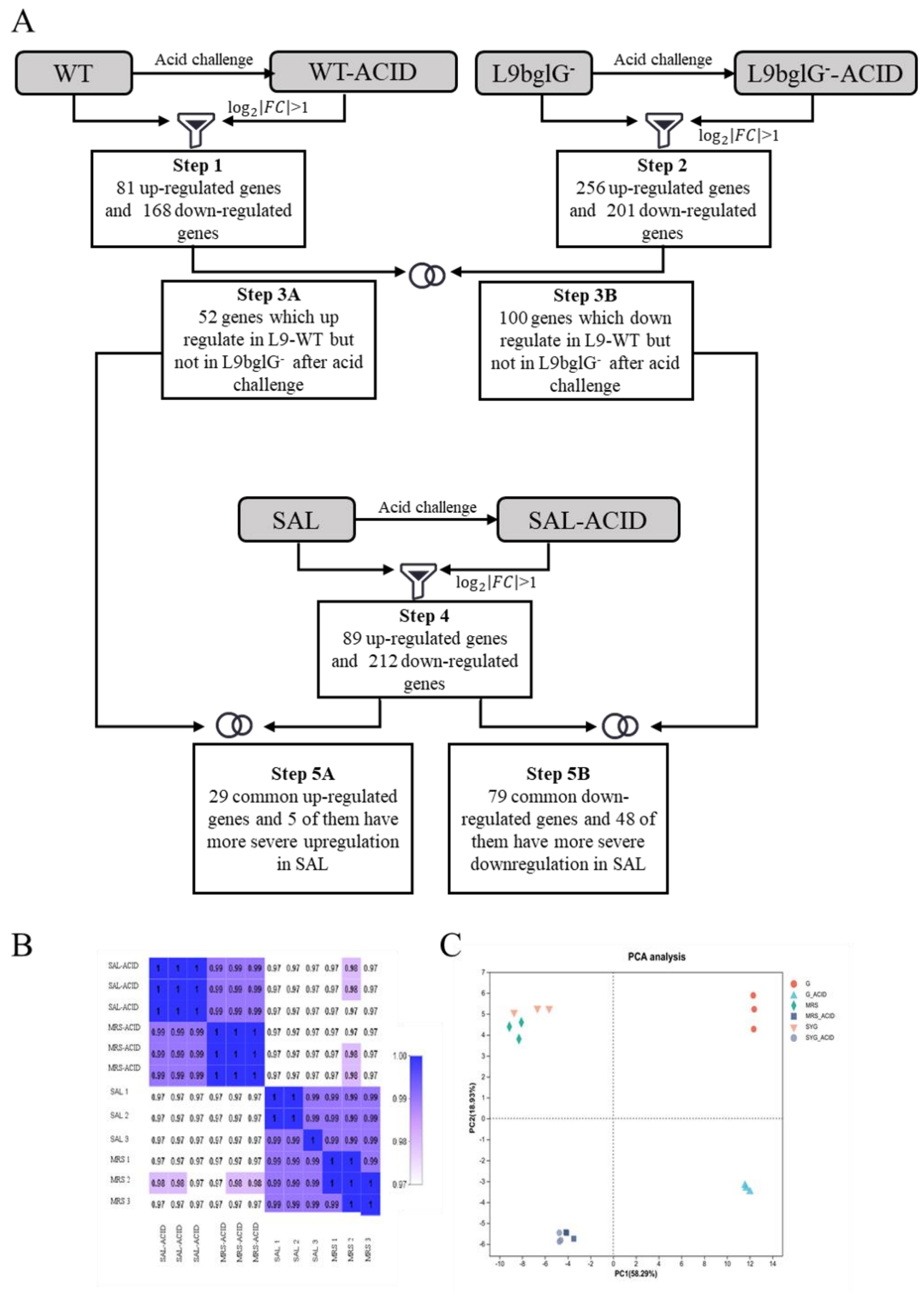

Figure S4. The analysis method of differential genes in ATR of *L. paracasei* L9 using RNA-Seq. The transcriptome data were screened and analyzed, and 108 genes showed different regulatory trends(**A**). Pearson correlation(**B**) and principal component analysis(**C**) between samples of RNA-Sequencing.

Table S1. Bacterial strains and plasmids.

Strains/Plasmids	Basic description	Source
Lacticaseibacillus paracasei L9	CGMCC No:9800	Ministry of Education Beijing Co-built Functional Dairy Laboratory, China
pUC19e	Amp^r pUC19 derived plasmid carrying Em resistance gene with independent promoter region	Ministry of Education Beijing Co-built Functional Dairy Laboratory, China
pUC*bgIG*	pUC19e carrying homologous sequence fragment of *bglG*	Constructed in the experiment
E.coli DH5a	Chemocompetent cells	Tiangen, China
$L9bglG^-$	Insertion inactivation mutant of *bglG*	Constructed in the experiment

Table S2. Primers used in PCR.

**Name**	**Primers(5′→3′)**
16s-F	GCACCGAGATTCAACATGG
16s-R	CTCACCAACTACTAATACCC
Q-*bglB*-F	GTAGTCGTAAGGTGATTGAT
Q-*bglB*-R	CAGAATTGGGAACAGAGA
Q-*bgIG*-F	CCGAGTATTGGTAGGCGTGTT
Q-*bgIG*-R	TCTTCCTGCGGTAATCGTTC
bglG-F	CCGGAATTCAAAGTGACATCGCCACAATC
bgIG-R	CGCGGATCCGCCAACAGCGATGAGTAAAG
T-*bglG*-F	CGGTGTTAGCCGAGATGAG
Em-R	AGCTTTGAACAATTCTTATCTCT

Table S3. 48 genes down-regulated in SAL & WT but not in L9bglG^{-}, had seriously downregulation in SAL.

gene ID	gene name	gene description	Log2FC (SAL_ACID /SAL)		Log2FC (WT_ACID /WT)
LPL9_RS14575	LPL9_RS14575	GNAT family N-acetyltransferase	-1.34	<	-1.04
LPL9_RS14330	LPL9_RS14330	iron-sulfur cluster biosynthesis family protein	-2.67	<	-1.96
LPL9_RS14320	cadA	cadmium-translocating P-type ATPase	-1.62	<	-1.29
LPL9_RS13195	LPL9_RS13195	YbhB/YbcL family Raf kinase inhibitor-like protein	-1.24	<	-1.19
LPL9_RS13030	LPL9_RS13030	NUDIX hydrolase	-2.22	<	-2.06
LPL9_RS12920	gltB	glutamate synthase large subunit	-3.58	<	-2.93
LPL9_RS12335	LPL9_RS12335	hypothetical protein	-1.54	<	-1.39
LPL9_RS12310	rpsN	30S ribosomal protein S14	-5.10	<	-4.87
LPL9_RS12300	LPL9_RS12300	metal ABC transporter ATP-binding protein	-3.72	<	-3.48
LPL9_RS12295	LPL9_RS12295	metal ABC transporter permease	-3.93	<	-3.49
LPL9_RS12290	LPL9_RS12290	zinc ABC transporter substrate-binding protein	-3.93	<	-3.64
LPL9_RS12275	LPL9_RS12275	hypothetical protein	-4.06	<	-3.36
LPL9_RS12265	LPL9_RS12265	MFS transporter	-1.60	<	-1.03
LPL9_RS12260	LPL9_RS12260	hypothetical protein	-3.05	<	-2.76
LPL9_RS12255	LPL9_RS12255	HAD-IC family P-type ATPase	-1.85	<	-1.42
LPL9_RS12250	LPL9_RS12250	Nramp family divalent metal transporter	-4.32	<	-3.57
LPL9_RS12240	LPL9_RS12240	hypothetical protein	-4.22	<	-3.76
LPL9_RS11985	LPL9_RS11985	hypothetical protein	-2.22	<	-1.61
LPL9_RS11955	LPL9_RS11955	low temperature requirement protein A	-1.30	<	-1.04
LPL9_RS11910	LPL9_RS11910	C1 family peptidase	-1.74	<	-1.63
LPL9_RS10050	LPL9_RS10050	iron-sulfur cluster biosynthesis family protein	-1.88	<	-1.23
LPL9_RS09815	LPL9_RS09815	alpha/beta hydrolase	-1.70	<	-1.23

LPL9_RS09560	LPL9_RS09560	ATP-binding cassette domain-containing protein	-1.91	<	-1.31
LPL9_RS09370	thiT	energy-coupled thiamine transporter ThiT	-1.06	<	-1.04
LPL9_RS09160	LPL9_RS09160	hypothetical protein	-1.63	<	-1.42
LPL9_RS09045	LPL9_RS09045	ring-cleaving dioxygenase	-1.22	<	-1.09
LPL9_RS08275	LPL9_RS08275	YxeA family protein	-2.24	<	-1.04
LPL9_RS08205	LPL9_RS08205	zinc-binding dehydrogenase	-2.24	<	-1.19
LPL9_RS07625	LPL9_RS07625	aldose 1-epimerase family protein	-1.64	<	-1.24
LPL9_RS07620	hslU	ATP-dependent protease ATPase subunit HslU	-2.05	<	-1.80
LPL9_RS07615	hslV	ATP-dependent protease subunit HslV	-2.02	<	-1.60
LPL9_RS07035	LPL9_RS07035	5-bromo-4-chloroindolyl phosphate hydrolysis family protein	-2.49	<	-1.52
LPL9_RS07030	LPL9_RS07030	toxic anion resistance protein	-2.46	<	-1.81
LPL9_RS06915	LPL9_RS06915	amino acid ABC transporter permease	-1.30	<	-1.20
LPL9_RS06660	LPL9_RS06660	hypothetical protein	-1.35	<	-1.03
LPL9_RS04000	nrdI	class Ib ribonucleoside-diphosphate reductase assembly flavoprotein NrdI	-1.04	<	-1.03
LPL9_RS03960	LPL9_RS03960	cation-translocating P-type ATPase	-1.23	<	-1.00
LPL9_RS03730	LPL9_RS03730	ABC transporter substrate-binding protein	-1.54	<	-1.05
LPL9_RS03695	LPL9_RS03695	hypothetical protein	-1.54	<	-1.26
LPL9_RS02595	LPL9_RS02595	homoserine O-succinyltransferase	-1.93	<	-1.14
LPL9_RS02435	LPL9_RS02435	dipeptide epimerase	-1.98	<	-1.69
LPL9_RS01010	LPL9_RS01010	GlsB/YeaQ/YmgE family stress response membrane protein	-1.27	<	-1.11
LPL9_RS00835	LPL9_RS00835	hypothetical protein	-1.29	<	-1.10
LPL9_RS00785	LPL9_RS00785	hypothetical protein	-1.54	<	-1.13
LPL9_RS00485	LPL9_RS00485	aspartate-semialdehyde dehydrogenase	-1.12	<	-1.02
LPL9_RS00480	LPL9_RS00480	diaminopimelate epimerase	-1.75	<	-1.64

LPL9_RS00185	LPL9_RS00185	Nramp family divalent metal transporter	-1.22	<	-1.21
LPL9_RS00170	LPL9_RS00170	TetR/AcrR family transcriptional regulator	-1.25	<	-1.08

Table S4. A total of 17 genes showed different regulatory trends between FCM-V subpopulation and FCM-I subpopulation and classified by COG Category.

Gene Name	COG Category	COG Type	COG Description	Annotated function	log2FC(FCM-V/Non-acid)	log2FC(FCM-I/Non-acid)
LPL9_RS10370	CELLULAR PROCESSES AND SIGNALING	Cell wall/membrane/envelope biogenesis	Nad-dependent epimerase dehydratase	Amino acid ABC transporter permease	2.0377 ↑	-1.0162 ↓
LPL9_RS03660	CELLULAR PROCESSES AND SIGNALING	Posttranslational modification, protein turnover, chaperones	response to heat	Hsp20/alpha crystallin family protein	-1.9645 ↓	1.2441 ↑
LPL9_RS14160	CELLULAR PROCESSES AND SIGNALING	Signal transduction mechanisms	PHOsphatase	NUDIX domain-containing protein	-1.7589 ↓	1.0555 ↑
LPL9_RS00270	INFORMATION STORAGE AND PROCESSING	Transcription	Transcriptional regulator	Transcriptional regulator	-3.3762 ↓	1.0407 ↑
LPL9_RS05810	INFORMATION STORAGE AND PROCESSING	Replication, recombination and repair	helicase	DEAD/DEAH boxhelicase	-1.0844 ↓	1.3583 ↑
LPL9_RS12000	METABOLISM	Amino acid transport and metabolism	Catalyzes the transfer of a phosphate group to glutamate to form glutamate 5-phosphate which rapidly cyclizes to 5-oxoproline (By similarity)	Glutamate 5-kinase	-2.7301 ↓	1.2204 ↑
LPL9_RS14325	METABOLISM	Inorganic ion transport and metabolism	Transporter	Hsp20/alpha crystallin family protein	-1.5069 ↓	1.2373 ↑
LPL9_RS00305	METABOLISM	Amino acid transport and metabolism	(ABC) transporter	ABC transporter permease	1.2064 ↑	-1.0832 ↓
LPL9_RS08205	METABOLISM	Energy production and conversion	Pyrophosphate phospho-hydrolase	Zinc-containing alcohol dehydrogenase/quinone oxidoreductase[NADPH]	-1.1713 ↓	1.0484 ↑
LPL9_RS14735	METABOLISM	Carbohydrate transport and metabolism	pts system	BglG family transcriptional antiterminator	1.0483 ↑	-1.1387 ↓
LPL9_RS07225	No details	No details	No details	Hypothetical protein	-1.4 ↓	1.0644 ↑
LPL9_RS00140	No details	No details	No details	Hypothetical protein	-1.1 ↓	1.9574 ↑

LPL9_RS08830	No details	No details	No details	Hypothetical protein	-2.8654 ↓	1.156 ↑
LPL9_RS10760	POORLY CHARACTERIZED	Function unknown	-	PTS beta-glucoside transporter subunit IIBCA	1.6958 ↑	-1.3129 ↓
LPL9_RS09420	POORLY CHARACTERIZED	Function unknown	acetyltransferase	N-acetyltransferase	-1.4664 ↓	1.1811 ↑
LPL9_RS03780	POORLY CHARACTERIZED	Function unknown	-	Iron-sulfur cluster biosynthesis family protein	1.275 ↑	-1.0363 ↓
LPL9_RS05775	POORLY CHARACTERIZED	Function unknown	ribonuclease BN	Flavodoxin	-1.2073 ↓	2.3676 ↑
